# Monoclonal Anti-Platelet Factor 4 Antibodies in Recurrent Pregnancy Loss

**DOI:** 10.1056/NEJMc2506014

**Published:** 2025-07-17

**Authors:** Natalie R. Bavli, Adam J. Kanack, Yoshito Nishimura, Noah P. Splinter, Emily E. Mauch, Alessandra J. Ainsworth, Ravi Sarode, Robert D. McBane, Mindy C. Kohlhagen, David Murray, Dong Chen, Rajiv K. Pruthi, Anand Padmanabhan

**Affiliations:** 1 University of Texas Southwestern Medical Center, Dallas, TX; 2 Mayo Clinic, Rochester, MN

## To the Editor:

Monoclonal anti-platelet factor 4 (PF4) antibodies have recently been shown to be a cause of persistent thrombotic thrombocytopenia^1–3^. We report two patients with monoclonal anti-PF4 antibody-mediated thrombosis, chronic-intermittent thrombocytopenia, and unexplained first-trimester pregnancy losses, suggesting a possible link between anti-PF4 antibodies and obstetric complications.

### Patient 1

**Figure 1.**
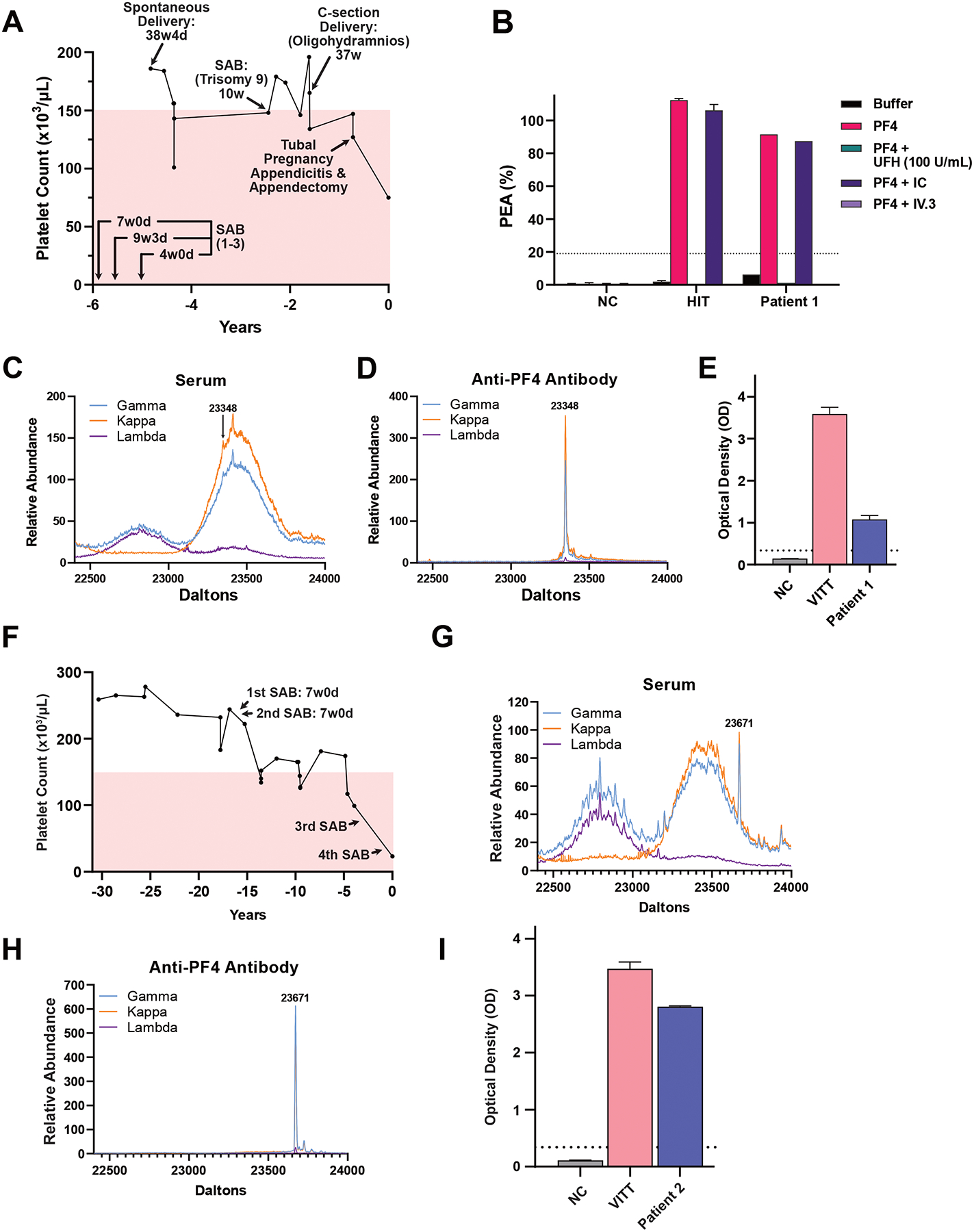
Clinical course, laboratory correlates, and research testing of the two patients. **(A)** The elapsed time from the first spontaneous abortion (SAB) is displayed. Available platelet counts are displayed as black circles. **(B)** PF4-dependent P-selectin expression (PEA) testing results are displayed for the patient’s sample in the following testing conditions: Buffer-treated platelets, PF4-treated platelets, PF4-treated platelets with high concentration of unfractionated heparin (UFH, 100 U/mL), PF4-treated platelets with an isotype control (IC) murine monoclonal antibody, or PF4-treated platelets with the FcgammaRIIa-blocking murine monoclonal antibody IV.3. PF4-dependent activation was inhibited by high concentrations of heparin and FcλRIIa blockade. NC- negative (healthy donor) control; HIT- HIT positive control. **(C)** Displayed are LC-ESI-QTOF MS light chain +11 distributions of the immunoglobulin light chains isolated from patient serum or **(D)** anti-PF4 antibodies affinity-purified from patient serum. Purple represents the distribution of all λ-containing Ig’s, orange represents the +11 m/z distribution of all κ-containing Ig’s, and blue represents the +11 m/z light chain distribution of all κ and λ light chains associated with an IgG heavy chain. The number listed above the peaks indicates the deconvoluted weight (Daltons) of the monoclonal anti-PF4 antibody. The x-axis shows the deconvoluted weight, and the y-axis shows the relative abundance of each identified peak. **(E)** Diagnostic testing results of a VITT ELISA utilizing uncomplexed PF4 as an antigenic target for Patient 1 (blue) are compared to the results from a negative (healthy donor) control sample (NC) or a known Ad26.COV2.S-associated VITT antibody (VITT). **(F)** Patient 2’s historical platelet counts until hospital admission are displayed as black circles. The pink overlay denotes platelet counts below the normal reference range (150 × 10^3^/μL). **(G)** The LC-ESI-QTOF MS light chain +11 distributions of the immunoglobulin light chains isolated from patient serum or **(H)** anti-PF4 antibodies affinity-purified from patient serum are displayed. Purple represents the distribution of all λ-containing Ig’s, orange represents the +11 m/z distribution of all κ-containing Ig’s and blue represents the +11 m/z light chain distribution of all κ and λ light chains associated with an IgG heavy chain. The number listed above the peaks indicates the deconvoluted weight (Daltons) of the monoclonal anti-PF4 antibody. The x-axis shows the deconvoluted weight, and the y-axis shows the relative abundance of each identified peak. **(I)** Diagnostic testing results of a VITT ELISA utilizing uncomplexed PF4 as an antigenic target for Patient 2 (blue) are compared to the results from a negative (healthy donor) control sample (NC) or a known Ad26.COV2.S-associated VITT antibody (VITT). LMWH - low molecular weight heparin; DTI - direct thrombin inhibitor; SAB- spontaneous abortion.

A 42-year-old G7P2 woman presented to the hospital with thrombocytopenia and unprovoked thrombosis (Figure S1) and developed additional thrombotic events despite anticoagulation. Heparin-induced thrombocytopenia (HIT) testing was positive in the enzyme-linked immunosorbent assay (ELISA, 0.759 optical density, OD), serotonin release assay (SRA; 81%), and PF4-dependent P-selectin expression assay^4^ (PEA; 89%). HIT serology has remained persistently positive over one year (Figure S1). Platelet counts were available over the preceding five years demonstrated chronic intermittent thrombocytopenia (Mean 151 × 10^9^/L; Range 59–196×10^9^/L; Figure 1A). During this period, she experienced three consecutive, unexplained, first-trimester pregnancy losses (7w0d, 10w3d, and 4w0d; Figure 1A).

Her serum induced PF4-dependent platelet activation that was inhibited by high concentrations of heparin and FcgammaRIIa blockade (Figure 1B), characteristic for anti-PF4 antibodies. IVIG antagonized platelet activation (Figure S2). Serum protein electrophoresis with immunofixation revealed no detectable monoclonal protein. Despite the absence of detectable monoclonal antibodies in serum by high-resolution mass spectrometry analysis (LC-ESI-QTOF MS; Figure 1C), monoclonal anti-PF4 antibodies were noted (Figure 1D). The antibodies bound uncomplexed PF4 in a vaccine-induced immune thrombotic thrombocytopenia (VITT)-specific ELISA (Figure 1E) and were negative in an automated latex immunoturbidometric assay (HemosIL HIT-Ab (PF4-H), consistent with VITT-like antibodies^5^.

### Patient 2

A 37-year-old G13P9 woman gave birth to a healthy neonate with brief exposure to low molecular weight heparin. Forty-two days postpartum, she developed a headache and thrombocytopenia with confirmed deep venous thrombosis and pulmonary embolism (Figure S3). She subsequently developed thrombosis of the portal, splenic, superior mesenteric, and right gonadal veins, complicated by small bowel ischemia requiring surgical resection. HIT ELISA and SRA testing were positive, consistent with an anti-PF4 antibody-mediated thrombosis (Figure S3). Thrombophilia workup was negative except for a weak anti-phosphatidylserine/prothrombin IgG (Table S2). HIT serology remained positive at the time of last assessment (>6 months since initial presentation; Figure S3).

Her obstetric history included 13 pregnancies: nine live births and four pregnancy losses. Full details of her pregnancy losses were unknown, although all were in the first trimester. Thrombocytopenia was first documented 15 years prior (130–140×10^9^/L; Figure 1F), with increasing frequency of thrombocytopenia over the preceding 10 years (Mean: 144×10^9^/L; Range: 99–181×10^9^/L). Similar to Patient 1, diagnostic testing was negative for monoclonal gammopathy of undetermined significance, MGUS. Despite negativity in the sensitive Mass-Fix technique, high-resolution mass spectrometry analysis of patient serum showed a small spike (Figure 1G) corresponding to a monoclonal anti-PF4 antibody (Figure 1H). Antibodies reacted in a VITT-specific ELISA (Figure 1I) and tested negative in the automated HemosIL HIT-Ab (PF4-H) assay, again supporting the presence of VITT-like antibodies.

These cases raise the possibility that persistent anti-PF4 antibodies may contribute to recurrent pregnancy loss, warranting further investigation in larger cohorts.

## Supplementary Material

Supplementary Appendix
