# Supplementary material for: Monoclonal Anti-Platelet Factor 4 Antibodies in Recurrent Pregnancy Loss
Source: N Engl J Med. Author manuscript; Available in PMC 2025 Jul 20. (PMC12276822; doi:10.1056/NEJMc2506014)
Supplement: Supplementary Appendix [file NIHMS2089883-supplement-Supplementary_Appendix.pdf]

Supplemental Appendix

Monoclonal anti-platelet factor 4 antibodies in recurrent pregnancy loss

Table of Contents

Figures……………………………………………………………………………………………..…2

Tables…………………………………………………………………………………………...….5

Detailed Patient histories...……………………………………………………………….....….6

Methods…………………………………………………………………………………..….…9

Acknowledgments…...………………………………………………………………………...….12

References……………………………………………………………………………………...….12

Figures

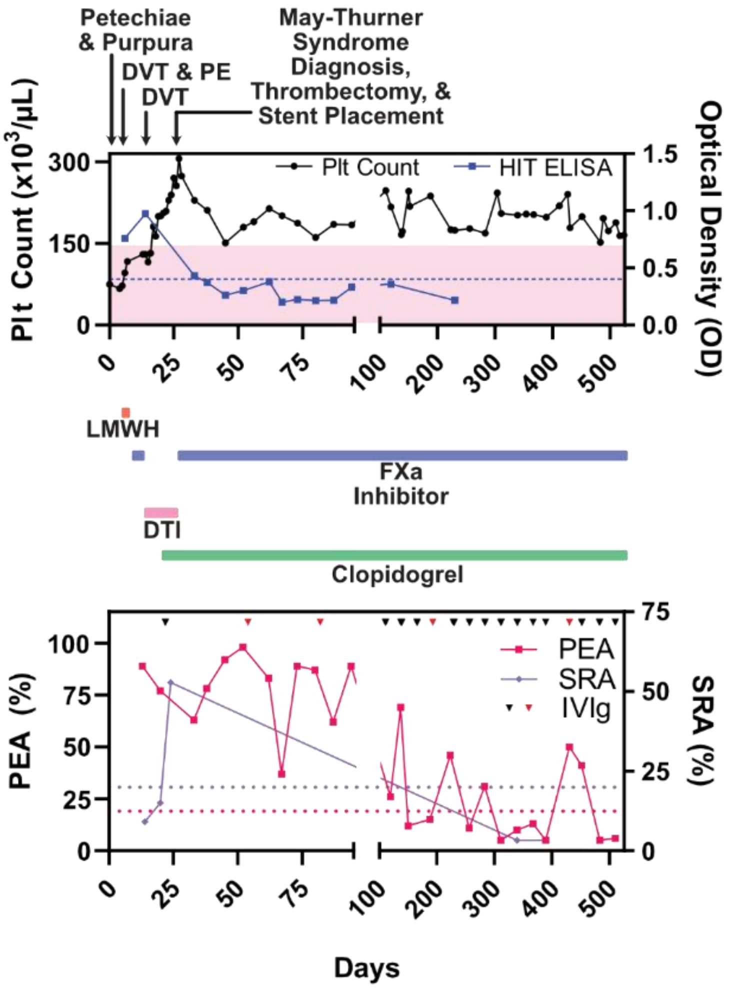

Supplementary Figure S1. Clinical course, laboratory correlates, and research testing in Patient 1. The elapsed time from the onset of petechia and purpura is displayed. Platelet counts are shown as black circles and correlate with the left Y-axis. The pink overlay denotes platelet counts below the normal reference range (150 × 10^3/µL). Blue squares represent HIT ELISA testing results and correlate with the right Y-axis. The dotted line represents the positive cut-off (0.4 OD) for HIT ELISA testing. Patient anticoagulation/antiplatelet medications are listed below the top graph. Functional platelet testing results and interventions with intravenous immunoglobulin (IVIg) are displayed in the bottom graph. Pink squares represent results of the PF4-dependent P-selectin expression assay (PEA) and correlate with the left Y-axis. Purple diamonds represent serotonin release assay (SRA) testing results and correlate with the right Y-axis. The pink and purple dashed line represent the positive cutoff for PEA and SRA testing, respectively. Single administrations of IVIg are represented by downward-facing red arrowheads, and administrations of IVIg on consecutive days are denoted by downward-facing black arrowheads.

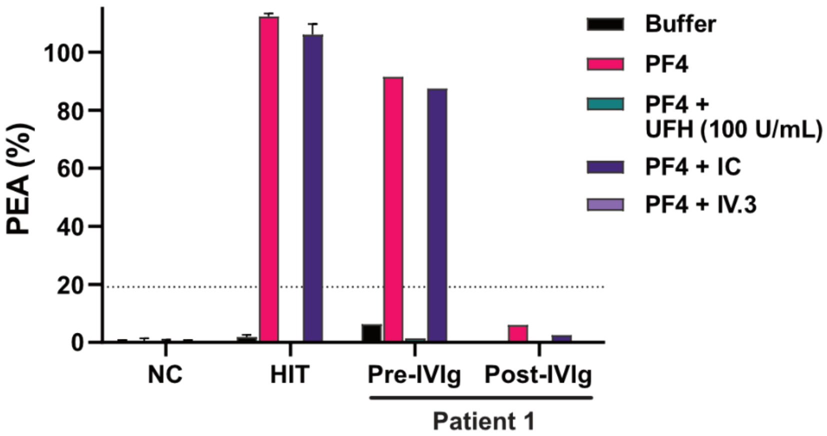

Supplementary Figure S2. PF4-dependent P-Selectin expression assay testing results for Patient 1. PEA testing results for Patient 1 are displayed for two samples obtained shortly before and after IVIg administration in the following testing conditions: Buffer-treated platelets, PF4-treated platelets, PF4-treated platelets with high concentration of unfractionated heparin (UFH, 100 U/mL), PF4-treated platelets with an isotype control (IC) murine monoclonal antibody, or PF4-treated platelets with the FcλRIIa-blocking murine monoclonal antibody IV.3. PF4-dependent activation was inhibited by both high concentrations of heparin and FcλRIIa blockade. NC- negative (healthy donor) control; HIT- HIT positive control.

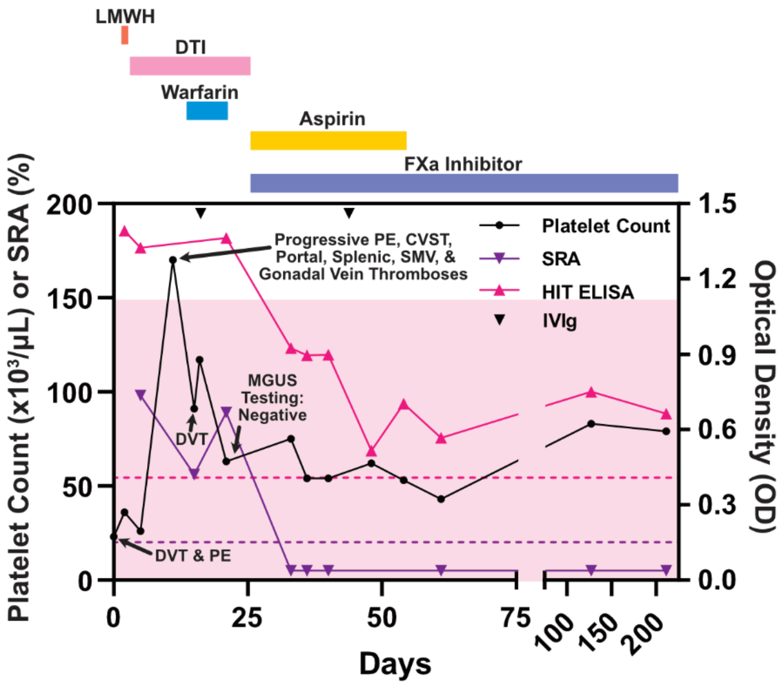

Supplementary Figure S3. Clinical course, laboratory correlates, and research testing in Patient 2. Patient anticoagulation/antiplatelet medications are listed above the top panel. The elapsed time from hospital admission for deep venous thrombosis and pulmonary embolism is displayed. Platelet counts are displayed as black circles and correlate with the left Y-axis. The pink overlay denotes platelet counts below the normal reference range (150 × 10^3/µL). Purple downward-facing arrowheads represent serotonin release assay (SRA) testing results and correlate with the left Y-axis. The purple dashed line represents the positive cut-off (20%) for SRA testing. Pink upward-facing arrowheads represent HIT ELISA testing results and correlate with the right Y-axis. The pink dashed line represents the positive cut-off (0.4 OD) for HIT ELISA testing. A downward-facing black arrowhead denotes the administration of intravenous immunoglobulin (IVIg) on consecutive days.

Tables.

Table S1

Results of thrombophilia testing in Patient 1.

Laboratory Test	Result	Reference Range
Dilute russell viper venom time screen	26.1 seconds	≤ 42.2 seconds
Silica clot time	38.8 seconds	≤ 44.9 seconds
Beta-2 glycoprotein 1 IgG, IgM and IgA	<9.4, 46 (repeat 12 weeks later <9.4), <9.4	≤ 40
Anticardiolipin IgG, IgM and IgA	<9.4, 24.2, <9.4	≤ 40
Antithrombin activity	79%	84 – 128%
Protein S activity	95%	83 – 138%
Protein S free antigen	67%	64 – 149%
Factor V Leiden mutation	Normal wild type	
Prothrombin mutation 20210G-A	Normal wild type	
Flow cytometry for paroxysmal nocturnal hemoglobinuria	Negative	Negative

Table S2.

Results of thrombophilia testing in Patient 2

Laboratory Testing	Result	Reference Range
Dilute russell viper venom time screen ratio	1.12	< 1.20
Beta-2 glycoprotein 1 IgG and IgM	<9.4, <9.4	< 15
Anticardiolipin IgG and IgM	<9.4, <9.4	< 15
Phosphatidylserine/Prothrombin Antibody, IgG and IgM	44.8, 24.8	< 30
Antithrombin activity	92%	80 – 130%
Protein S activity	88%	70 – 150%
Protein S free antigen	74%	50 – 160%
Activated Protein C Resistance V	2.5	≥ 2.3
Prothrombin mutation 20210G-A	Normal wild type	

Detailed patient histories

Patient 1

A 42-year-old G7P2 woman presented with purpura, petechiae, and thrombocytopenia, unprovoked occlusive left posterior tibial vein thrombosis, and bilateral pulmonary emboli. She was treated with one dose of enoxaparin and transitioned to apixaban before discharge from the hospital (10 mg twice daily for seven days, followed by 5 mg twice daily). She returned a week later with worsening left lower extremity swelling, with imaging showing a new proximal deep vein thrombosis (DVT) in the left common femoral vein with extension into the common iliac vein and was confirmed to have May-Thurner syndrome despite full compliance with apixaban. D-dimer was elevated at 2.29 mg/L FEU (reference range ≤0.59 mg/L FEU) and heparin-induced thrombocytopenia (HIT) testing was positive in the enzyme-linked immunosorbent assay (ELISA, 0.759 optical density, OD), serotonin release assay (SRA; 81%), and PF4-dependent P-selectin expression assay[11] (PEA; 89%) but negative in in an automated latex immunoturbidometric assay (HemosIL HIT-Ab (PF4-h), reminiscent of VITT. Thrombophilia testing, including antiphospholipid syndrome, was unrevealing except for a borderline decrease in antithrombin activity. She was treated with bivalirudin, followed by intravenous immune globulin (IVIG; 2 g/kg). She underwent left iliofemoral venous thrombectomy, stenting of the left common and external iliac vein, and venoplasty of the left common femoral vein. Due to persistent positive results in the PEA and concern for recurrent thrombosis in the setting of stent placement, she was continued on apixaban and clopidogrel, in addition to monthly IVIG (2 g/kg given over two consecutive days), without further recurrence of thrombosis. PEA results remain persistently positive even one year after the acute thrombotic event.

Platelet counts were available for the preceding five years and demonstrated chronic intermittent thrombocytopenia, with decreased or borderline-normal values (Mean 151 x 10^9/L; Range 59-196x10^9/L). During this time, she had seven pregnancies and two live births (G7P2).

She had three consecutive, unexplained, first trimester losses (7w0d, 10w3d, and 4w0d), one genetically abnormal pregnancy loss at 10w9d (trisomy 9) and one ectopic pregnancy. Her 4th and 6th pregnancies resulted in live birth; however, both were complicated by hypertensive disease of pregnancy at term. Her 4th pregnancy was delivered at 38w0d. Placental pathology revealed a size smaller than expected for gestational age, with paramarginal cord insertion, and distal villous hyperplasia. Her 6th pregnancy was delivered at 37w0d and complicated by oligohydramnios. Placental pathology was notable for intramural fibrin deposition in the chorionic plate vessels, patchy villous edema, and acute subchorionitis.

Serum protein electrophoresis with immunofixation revealed no detectable monoclonal protein to support a diagnosis of monoclonal gammopathy of undetermined significance (MGUS). Bone marrow examination was unremarkable, with no morphologic or immunophenotypic evidence of hematolymphoid malignancy, and the myeloma FISH panel was normal. The patient did not have hypothyroidism or uterine structural defects. The patient and her partner had normal chromosomal analysis, and her partner was also tested for retrograde ejaculation and semen volume, both of which were normal.

Patient 2

A 37-year-old G13P9 woman gave birth to a healthy neonate at 39 weeks without immediate postpartum complications, with brief exposure to low molecular weight heparin during the postpartum period for DVT prophylaxis. Forty-two days postpartum, she presented to the emergency department with a headache and thrombocytopenia ($55x10^9$/L). She was discharged with symptomatic management. Ten days later, she returned with bilateral lower extremity swelling and dyspnea. Laboratory evaluation revealed severe thrombocytopenia (23×10^9/L). D-dimer on presentation was considerably elevated to 91,006 ng/mL (reference range, $\leq$500 ng/mL). Imaging confirmed an acute right lower extremity deep vein thrombosis with concurrent segmental and subsegmental pulmonary emboli and splenomegaly, and she was started on therapeutic dose intravenous unfractionated heparin.

On hospital day five, she developed severe abdominal pain, and imaging revealed thrombosis of the portal, splenic, superior mesenteric and right gonadal veins complicated by small bowel ischemia which required surgical resection. HIT ELISA and SRA testing were positive, consistent with an anti-PF4 antibody-mediated thrombotic syndrome, but negative in the automated HemosIL HIT-Ab (PF4-H) test, consistent with a VITT-like antibody. Except for weak-positive antinuclear antibody (1:160) and anti-phosphatidylserine/prothrombin immunoglobulin G (IgG) antibody (44.8U; reference range: <40U, a non-criteria antibody for antiphospholipid antibody syndrome), the thrombophilia workup was negative. Heparin was immediately discontinued, and she was initiated on bivalirudin and IVIG (2g/kg). During attempts to transition to warfarin, she experienced recurrent thrombocytopenia (platelet nadir: $91x10^9/L$) and developed progression of the right lower extremity DVT requiring an additional course of IVIG (2g/kg). She was transitioned from bivalirudin to apixaban and aspirin. With no further thrombotic events, she was discharged on hospital day 29. While SRA testing became negative 34 days after hospitalization, HIT ELISA has remained positive for >6 months. The patient is currently being maintained on apixaban.

The patient's obstetric history included 13 pregnancies: nine live births and four pregnancy losses (G13P9). All live births were delivered vaginally, at term. Her 5th pregnancy was complicated by neonatal gastroschisis. Full details of her pregnancy losses are unknown, although all were in the first trimester and occurred between her live births. She was noted to be mildly thrombocytopenic 15 years before presentation ($130\text{-}140x10^9/L$), with increased frequency of thrombocytopenia in the prior 10 years (Mean: $144x10^9/L$; Range: $99\text{-}181x10^9/L$). The patient's chronic intermittent thrombocytopenia suggested the potential presence of a persistent anti-PF4 antibody-mediated process. Similar to Patient 1, diagnostic testing was negative for MGUS (tested by the sensitive Mass-Fix technique[13]). The patient did not have hypothyroidism or uterine structural defects. She did not have any genetic testing results available in her medical records.

Methods

Patient samples

Blood samples were obtained from patients after informed consent. The Mayo Clinic Institutional Review Board approved all research studies.

Anti-PF4 antibody isolation

Anti-PF4 antibodies were isolated as described recently[1,2]. Briefly, heparin sepharose beads (200 μL, Cytiva Lifesciences) were washed with phosphate-buffered saline, pH 7.4 (PBS), and incubated with 200 μL (1 mg/mL) of recombinant PF4 (Protein Foundry) for one hour. Beads were then incubated with 250 μL of patient sample (serum) for one hour. Beads were washed with PBS, and elution from the PF4/heparin sepharose beads was performed with 250 μL of 2M NaCl. Eluates were dialyzed against PBS before evaluation by ELISA, functional platelet assays, or mass spectrometric studies.

Functional platelet studies

The SRA was performed in various CLIA-approved reference testing laboratories, as determined by the treating physician. The PEA was performed as previously described[3,4]. Prostaglandin E1 was added to citrated whole blood from healthy volunteers at 50 ng/mL before centrifugation at 200 x g for 15 minutes to obtain platelet-rich plasma (PRP). Platelets were isolated from PRP by secondary centrifugation at 1,000 x g for 15 minutes. Platelets were then resuspended in phosphate-buffered isotonic saline (PBS) supplemented with 1% bovine serum albumin at pH 7.4. Platelets (1×10^6) were treated for 20 minutes at room temperature using the following conditions: Buffer-treated platelets, PF4-treated platelets (37.5 μg/mL PF4), PF4-treated platelets with high concentration of unfractionated heparin (37.5 μg/mL PF4 + UFH, 100 U/mL), PF4-treated platelets with an isotype control (IC) murine monoclonal antibody (37.5 μg/mL PF4 + 2 μg/mL murine isotype control monoclonal antibody), or PF4-treated platelets with

the FcλRIIa-blocking murine monoclonal antibody IV.3. with (37.5 μg/mL PF4 + 2 μg/mL IV.3 murine monoclonal antibody). After PF4 incubation, ten microliters of patient sample were added to 40 μL of PF4-treated platelets and incubated for one hour at room temperature, yielding a final PF4 concentration of 30 μg/mL. Fluorescently labeled anti-P-selectin (monoclonal antibody HB-299, ATCC) and anti-GPIIIa (monoclonal antibody HB-242, ATCC) antibodies were added to the samples for 20 minutes before being analyzed by flow cytometry. Platelet events were gated for GPIIIa positivity in flow cytometry, and P-selectin expression (median fluorescence intensity, MFI) was recorded.

Liquid Chromatography Electrospray Ionization Quadrupole time-of-flight mass spectrometry (LC-ESI-QTOF MS)

The basic method used for antibody analysis has been previously described[5,6]. Immunoglobulins (Igs) from patient sera or bead eluates were isolated using camelid-derived nanobodies directed against the constant domains of human Ig gamma heavy chain, kappa light chain, or lambda light chains (Thermo Fisher Scientific). For each pulldown, 100 μL or 50 μL of camelid nanobody beads were incubated with 10 μL of serum or 100 μL of PF4-Heparin Sepharose eluate, respectively, diluted into 200 μL of PBS and incubated for 30 minutes at ambient temperature. Supernatants were removed, and the nanobody-linked beads were washed three times with 500 μL of water. Bound immunoglobulins (Igs) from serum or anti-PF4 antibody eluates were eluted using 60μL or 20μL of 5% acetic acid. After five minutes, the eluted Igs were reduced using dithiothreitol (DTT) in 1M ammonium bicarbonate (2:1; v:v) to disassociate immunoglobulins and separate light chain from heavy chain Ig components. An Agilent 1290 Infinity II liquid chromatography (LC) system was used to separate immunoglobulin chains before ionization and to reduce PF4 background before analysis using a SCIEX Zeno time-of-flight (TOF) 7600 mass spectrometer (MS). Ten microliters of each camelid nanobody bead eluate was injected per analysis onto a Poroshell 300SB-C3 column (2.1 mm X 75 mm)

with a five µm particle size placed in a 60 ºC column heater. The mobile phases included an aqueous phase A (100% water + 1% formic acid) and an organic phase B (90% acetonitrile + 10% isopropanol + 0.1% formic acid), and the flow rate was 300 µL/min. The diverter valve was used to direct 10.35 minutes of the gradient into the MS; otherwise, the LC was diverted to waste. The MS, using positive electrospray ionization, was run using intact protein workflow; CUR 30, CAD 7 GAS1 35, GAS2 30, and temperature 500 °C. TOF MS data was collected from 600 to 2500 m/z; DP 175 and CE 10. Data analysis was performed using Analyst TF v1.8.1 and PeakView ver. 2.2. The presence of immunoglobulins was defined from the light chain +11 (m/z, mass to charge 2020 to m/z 2200) as described elsewhere[5,6]. The retention time of the monoclonal light chain in each patient sample was tracked using PeakView. The mass spectra of the multiply charged light chain ions were deconvoluted to obtain an accurate molecular mass using the Bio Tool Kit ver. 2.2 plug-in software. The instrument was calibrated every five samples using an automated calibrant delivery system (CDS). Mass measurement accuracy was estimated to be 15 ppm for the duration of the analysis.

HIT ELISA

Patient samples were evaluated in Lifecodes PF4 IgG (Immucor) ELISA, an FDA-approved in vitro diagnostic assay that employs PF4-polyvinyl sulfonate (PVS) targets. Lifecodes PF4 IgG (Immucor) was used according to the manufacturer’s instructions. In brief, patient serum/plasma was incubated with PF4-PVS coated wells and extensively washed. Each well was then incubated with an alkaline phosphatase-labeled anti-human IgG antibody. After secondary antibody incubation, pNPP substrate was added, and colorimetric detection was performed.

VITT ELISA

Testing for VITT/VITT-like anti-PF4 antibodies was performed using uncomplexed, chemically cross-linked recombinant PF4. Cross-linking of PF4 was carried out by combining 1 mg/mL EDC

and recombinant PF4 at an equal ratio (v:v) in 15 mM MES buffer pH 8.0 for six hours at ambient temperature. Cross-linking reactions were stopped by adding 1M Tris pH 7.4 buffer at a 1:4 (v:v) ratio of the cross-linked PF4 polypeptide reaction to Tris buffer. The VITT ELISA was completed by immobilizing uncomplexed cross-linked PF4 (0.5 µg/well) on ELISA plates (Thermo Scientific) overnight at 4°C. ELISA plates were washed three times with PBS pH 7.4 +0.1% TWEEN®-20 and blocked with SUPERBLOCK T20 (Thermo Scientific). Patient samples were added to the plate at 1:50 dilution for one hour, followed by four washes with PBS/0.1% TWEEN®-20. After a 45-minute incubation with 50 µL of alkaline phosphatase-conjugated goat anti-human IgG Fc antibody (Jackson Immunoresearch) at a dilution of 1:5000, four additional washes were performed using PBS/0.1% TWEEN®-20. Colorimetric detection was then performed using p-nitrophenyl phosphate (pNPP) substrate, and the optical density (OD; 405 nm minus 492 nm) at 30 minutes was recorded. The positive cut-off of the VITT ELISA was set at three standard deviations above the mean optical density of 50 healthy donor samples (0.341).

Acknowledgments

We acknowledge Lisa Hines for exceptional research coordination support.

References

1. Kanack AJ, Schaefer JK, Sridharan M, et al. Monoclonal gammopathy of thrombotic/thrombocytopenic significance. Blood 2023;141:1772-6.
2. Kanack AJ, Bayas A, George G, et al. Monoclonal and oligoclonal anti-platelet factor 4 antibodies mediate VITT. Blood 2022;140:73-7.
3. Padmanabhan A, Jones CG, Curtis BR, et al. A Novel PF4-Dependent Platelet Activation Assay Identifies Patients Likely to Have Heparin-Induced Thrombocytopenia/Thrombosis. Chest 2016;150:506-15.
4. Samuelson Bannow B, Warad DM, Jones CG, et al. A prospective, blinded study of a PF4-dependent assay for HIT diagnosis. Blood 2021;137:1082-9.
5. Barnidge DR, Dasari S, Ramirez-Alvarado M, et al. Phenotyping polyclonal kappa and lambda light chain molecular mass distributions in patient serum using mass spectrometry. J Proteome Res 2014;13:5198-205.
6. Barnidge DR, Dasari S, Botz CM, et al. Using mass spectrometry to monitor monoclonal immunoglobulins in patients with a monoclonal gammopathy. J Proteome Res 2014;13:1419-27.
